# Using artificial intelligence to reduce diagnostic workload without compromising detection of urinary tract infections

**DOI:** 10.1186/s12911-019-0878-9

**Published:** 2019-08-23

**Authors:** Ross J. Burton, Mahableshwar Albur, Matthias Eberl, Simone M. Cuff

**Affiliations:** 1 Department of Infection Sciences, Severn Pathology, Bristol, BS10 5NB UK; 2 Division of Infection and Immunity, School of Medicine, Cardiff University, Henry Wellcome Building, Heath Park, Cardiff, CF14 4XN UK; 3 Systems Immunity Research Institute, Cardiff University, Heath Park, Cardiff, CF14 4XN UK

**Keywords:** Urinary tract infection, Machine learning, Laboratory medicine, Algorithms, Diagnostic decision making

## Abstract

**Background:**

A substantial proportion of microbiological screening in diagnostic laboratories is due to suspected urinary tract infections (UTIs), yet approximately two thirds of urine samples typically yield negative culture results. By reducing the number of query samples to be cultured and enabling diagnostic services to concentrate on those in which there are true microbial infections, a significant improvement in efficiency of the service is possible.

**Methodology:**

Screening process for urine samples prior to culture was modelled in a single clinical microbiology laboratory covering three hospitals and community services across Bristol and Bath, UK. Retrospective analysis of all urine microscopy, culture, and sensitivity reports over one year was used to compare two methods of classification: a heuristic model using a combination of white blood cell count and bacterial count, and a machine learning approach testing three algorithms (Random Forest, Neural Network, Extreme Gradient Boosting) whilst factoring in independent variables including demographics, historical urine culture results, and clinical details provided with the specimen.

**Results:**

A total of 212,554 urine reports were analysed. Initial findings demonstrated the potential for using machine learning algorithms, which outperformed the heuristic model in terms of relative workload reduction achieved at a classification sensitivity > 95%. Upon further analysis of classification sensitivity of subpopulations, we concluded that samples from pregnant patients and children (age 11 or younger) require independent evaluation. First the removal of pregnant patients and children from the classification process was investigated but this diminished the workload reduction achieved. The optimal solution was found to be three Extreme Gradient Boosting algorithms, trained independently for the classification of pregnant patients, children, and then all other patients. When combined, this system granted a relative workload reduction of 41% and a sensitivity of 95% for each of the stratified patient groups.

**Conclusion:**

Based on the considerable time and cost savings achieved, without compromising the diagnostic performance, the heuristic model was successfully implemented in routine clinical practice in the diagnostic laboratory at Severn Pathology, Bristol. Our work shows the potential application of supervised machine learning models in improving service efficiency at a time when demand often surpasses resources of public healthcare providers.

**Electronic supplementary material:**

The online version of this article (10.1186/s12911-019-0878-9) contains supplementary material, which is available to authorized users.

## Background

For routine clinical microbiology diagnostic laboratories, the highest workload is generated by urine samples from patients with suspected urinary tract infection (UTI) [1]. According to the UK Standards of Microbiological Investigations, UTIs are defined as the ‘presence and multiplication of microorganisms, in one or more structures of the urinary tract, with associated tissue invasion’. The most common causative pathogen is *E. coli* followed by other members of the *Enterobacteriaceae* family. The incidence of UTIs varies with age, gender, and comorbidities. Women experience a higher incidence than men, with 10–20% suffering from at least one symptomatic UTI throughout their lifetime. Most UTIs that occur in men are associated to physiological abnormalities of the urinary tract. In children, UTIs are common but often difficult to diagnose due to non-specific symptoms. Where a UTI is suspected, a urine sample is collected for processing by a centralised diagnostic laboratory. Upon arrival, the sample receives microscopic analysis, microbiological culture, and where necessary, antimicrobial sensitivity testing [2]. However, many urine samples will yield a negative culture result, no significant bacterial isolate or mixed culture results suggesting sample contamination. Such ambiguous and diagnostically unhelpful outcomes typically occur in approximately 70–80% of urine samples cultured [3–8]. This creates opportunities for significant cost savings. At the same time, diagnostic microbiology laboratories in the UK and elsewhere are undergoing transition to full laboratory automation [9–11]. With a view to assist with the consolidation of services [12] and changes in laboratory practice, appropriate pre-processing and classification of urine samples prior to culture might be required to reduce the number of unnecessary cultures performed.

In many hospitals, automated urine microscopy is performed prior to culture using automated urine sediment analysers. This is a common precursor to culture and informs on the cellular content of the urine sample, where evidence of pyuria results in direct antimicrobial sensitivity testing accompanying culture; in addition to culture on chromogenic agar, urine is applied directly to nutrient agar for sensitivity testing by Kirby–Bauer method. The use of microscopic analysis, biochemical dip-stick testing, and flow cytometry for predicting urinary tract infection are well documented in the literature. The current consensus is that WBC count and bacterial count correlate with culture outcome [3, 4, 13] but not well enough to replace culture entirely. We here explored the potential for a machine learning solution to reduce the burden of culturing the large number of culture-negative samples without reducing detection of culture-positive samples, with concessions made for particularly vulnerable patient groups.

We speculated that the application of a statistical machine learning model that accounts not just for current diagnostic results but also for historical culture outcome, as well as clinical details and demographical data, could potentially reduce laboratory workload without compromising the detection of UTIs. We contrast the classification performance of heuristic microscopy thresholds with three machine learning algorithms: A Random Forest classifier, a Neural Network with a single hidden layer, and the Extreme Gradient Boosting algorithm XGBoost. Random Forest classifiers are one of many ensemble methods, where the predictions of multiple base estimators are used to improve classification. In a Random Forest multiple ‘trees’ are constructed, each from a bootstrap sample of the training data and a random subset of features. The resulting classification is a result of the average of all the ‘trees’, hence the name ‘Random Forest’ [14]. Neural Networks are supervised learning algorithms made up of multiple layers of ‘perceptrons’ with assigned weights, which when summed and provided to a step function, produce a classification output. By optimising a loss function and adjusting the weights through a process called ‘backpropagation’, Neural Networks can learn non-linear relationships [14]. Boosting algorithms, such as the XGBoost algorithm in this study, generate a decision tree using a sample of the training data. The performance of the trained classifier, when tested using all the training data, is used to generate sample weights that influence the next classifier. An iterative process then occurs, each time generating a new classifier that is informed by the misclassification of the prior classifier [15].

## Methods

### Patient samples and data pre-processing

This project was performed as part of a service improvement measure on anonymised retrospective data at Southmead Hospital Bristol, North Bristol NHS Trust, UK, and was approved locally by the service manager and head of department. Urine samples with specimen date between 1st October 2016 and 1st October 2017 (*n* = 225,207) were extracted from the Severn Pathology infectious science services laboratory information management system (LIMS), Winpath Enterprise. Additional file 2: Figure S1 details pre-processing steps taken prior to investigation of microscopy thresholds and machine learning algorithms. Samples that received manual microscopy (often due to excessive haematuria or pyuria) and those from catheterised patients were excluded from the study. All preprocessing was performed in the Python programming language (version 3.5) utilising the Pandas library (version 0.23). The dependent variable, the culture result, was classified using regular expression to create a binary outcome; positive outcome was denoted as any significant bacterial isolate with accompanying antimicrobial sensitivities, whereas a negative outcome was a culture result of ‘no growth’, ‘no significant growth’, or ‘mixed growth’.

Microscopy counts for white blood cells (WBCs) and red blood cells (RBC) were artificially capped at 100/μl due to the interface between SediMAX and Winpath Enterprise implemented in the laboratory. For the same reason, epithelial cell count was capped at 25/μl. No adjustments are made here as the data set represents ‘real-world’ data and the type of data a model would encounter in practice. The bacterial cell count was heavily positively skewed. To counteract the effect of outliers without deviating from a representation of typical data, bacterial counts that exceeded the 0·99 percentile were classed as outliers and removed. Two additional features were engineered from the microscopy cell counts: ‘haematuria with no WBCs’ and ‘pyuria with no RBCs’. Pyuria was defined as a WBC count > = 10/μl and haematuria as ≥3/μl, as described in the UK Standards for Microbiology Investigations [12].

### Patient groupings by clinical indicators

We defined several significant patient groups with a higher incidence of UTI based on clinical advice and prior published work [2, 16, 17]. For each of these groups we created a list of keywords for association (Additional file 1: Table S1). Using the Levenshtein distance algorithm implemented in the Natural Language Toolkit library (NLTK, version 3.3) [18] with an edit distance threshold of one or less, keywords were compared to clinical details provided with urine specimens, to classify specimens into significant patient groups. This implementation was chosen to negate errors in spelling and grammar in the clinical details provided, and as a result of its ease of use and popularity in text mining and bioinformatics applications [18, 19].

To increase the accuracy of patient grouping, clinical details were consolidated where multiple samples were received from the same patient; approximately 58% of patients in the data set studied had multiple samples. For acute kidney infection, occurrence of keywords within a two-week timeframe resulted in allocation of a patient to this group. In the case of pregnancy, this timeframe was increased to nine months. When allocating patients to the pre-operative group, only the clinical details unique to a sample were considered. For all other groups the assumption was made that conditions are chronic and keyword search was conducted on the consolidation of all clinical details.

Using the same methodology as the patient grouping, two additional variables were engineered from the clinical details: the reported presence of nitrates in the urine and descriptive qualities of the sample such offensive smell and/or appearance.

### Exploratory data analysis and implementation of heuristic models and machine learning algorithms

Heuristic models using microscopy thresholds, as well as the machine learning algorithms, were developed in the Python programming language (version 3.5) utilising the Pandas (version 0.23) [20] and Sci-kit learn (version 0.19) [14] libraries. Exploratory data analysis was performed in R (version 3.4.3) utilising the TidyVerse packages (version 1.2.1) [21] and base functions. Data visualisation and graphical plots were created using the Python library Seaborn (version 0.9.0) [22]. Three machine learning algorithms were assessed: multi-layer feed-forward Neural Network, Random Forest Classifier, and XGBoost Gradient Boosted Tree Classifier. Random Forests, Neural Networks, and Boosting Ensembles have been noted as having the best performance in terms of accuracy amongst 17 ‘families’ investigated [23]. Data was randomly split into training (70%, *n* = 157,645) and holdout data (30%, *n* = 67,562). Holdout data was used for model validation. Model training and parameter optimisation was performed using a grid-search algorithm with k-fold (k = 10) cross-validation, where the model parameters where chosen based on area under receiver operator curve (AUC Score). Performance of models were measured as a balance between classification sensitivity and relative workload reduction when tested on holdout data; classification sensitivity took precedent in the choice of model, but once an optimal sensitivity of 95% was met, workload reduction was the deciding metric. Classification sensitivity and specificity were calculated as described in Additional file 3: Figure S2. 95% confidence intervals were calculated using the normal approximation method. Due to the size of the data-set studied and following guidance published by Raschka S [24], the Cochran’s Q test was selected to formally test for statistically significant difference in accuracy amongst models (*p* <  0.05). Where this condition is met, the McNemar test was used post hoc for individual model comparison with Bonferroni’s correction for multiple comparisons; McNemar and Cochran’s Q test implemented using the MLXtend python library [25].

## Results

### Patient characteristics

Around 20% of the samples in the data belonged to inpatients, with an incidence of significant culture of 20·8% (Table 1). The ratio of female to males was approximately 3:1, but the incidence of significant culture was similar with 21·6% and 26·8% for males and females, respectively. Amongst the groupings generated from clinical details ‘Pregnant’ and ‘Persistent/Recurrent Infection’ contributed to the largest proportion of the overall data, with all other groups consisting of less than 12% of the data set. Samples categorised as ‘Persistent/Recurrent Infection’ showed an incidence of significant growth of almost 40%. The small number of samples whose clinical details included offensive smell or testing positive for nitrates showed the highest incidence of significant culture. Additionally, the presence of pyuria in the absence of red blood cells, a condition reported in 11·6% of samples, showed in excess of 50% bacterial culture-positive results. The age distribution for female patients was multimodal, with a peak between 20- and 40-years accounting for the pregnant women (Additional file 4: Figure S3). For males, the distribution was bimodal, with most samples coming from elderly individuals.

**Table 1:** Description of categorical variables

	*n*	Proportion of entire dataset (%)	Incidence of significant bacterial growth (%)	Variance
Positive culture	57,857	27·19		
Negative culture	154,771	72·81		
Patient groups				
Persistent/recurrent infection	47,348	22·28	37·68	0·17
Pregnant	28,222	13·28	7·16	0·12
Renal inpatient/outpatient	11,755	5·55	26·20	0·05
Pre-operative patient	9463	4·45	21·84	0·04
Acute kidney disease	3891	1·83	31·23	0·02
Immunocompromised	2114	0·66	23·18	0·01
Multiple Sclerosis	1046	0·49	24·38	0·005
Inpatient	43,349	20·40	20·81	0·16
Positive for nitrates	5895	2·80	59·73	0·03
Offensive smell	270	0·10	55·19	0·001
Pyuria, no RBCs	24,587	11·60	52·27	0·10
Haematuria, no WBCs	368	0·002	0·06	0·002
Age				
< 11 years old	14,594	6·87	17·23	
Gender				
Males	54,070	25·40	21·58	
Females (total)	158,422	74·60	26·76	
Females (not pregnant)	130,200	61·29	33·85	

### Exploratory data analysis

**Fig. 1:**
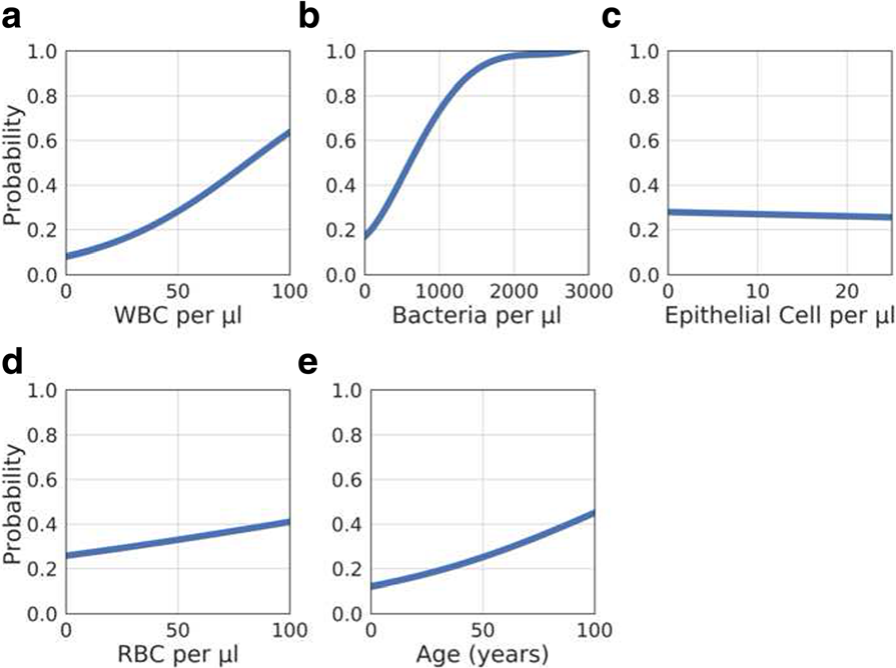
5th Order Polynomial describing the probability of a significant bacterial culture result as determined by logistic regression, in relation to **a** WBC counts, **b** RBC counts, **c** Age, **d** epithelial cell counts, and **e** bacterial counts

Exploratory data analysis revealed that among the four microscopic cell counts performed, WBC and bacterial counts per μl showed the strongest correlation with the probability of significant bacterial growth on culture (Fig. 1). RBC and epithelial cell count were not significantly associated with culture outcome. To confirm the relationships observed in Fig. 1, an individual Logistic Regression model trained using cellular counts showed that inclusion of WBC and bacterial counts exhibited a higher reduction in residual deviance when compared to RBC and epithelial cell count. Age of the patient also positively correlated with the probability of significant growth, albeit to a lesser extent when compared to WBC and bacterial counts.

**Fig. 2:**
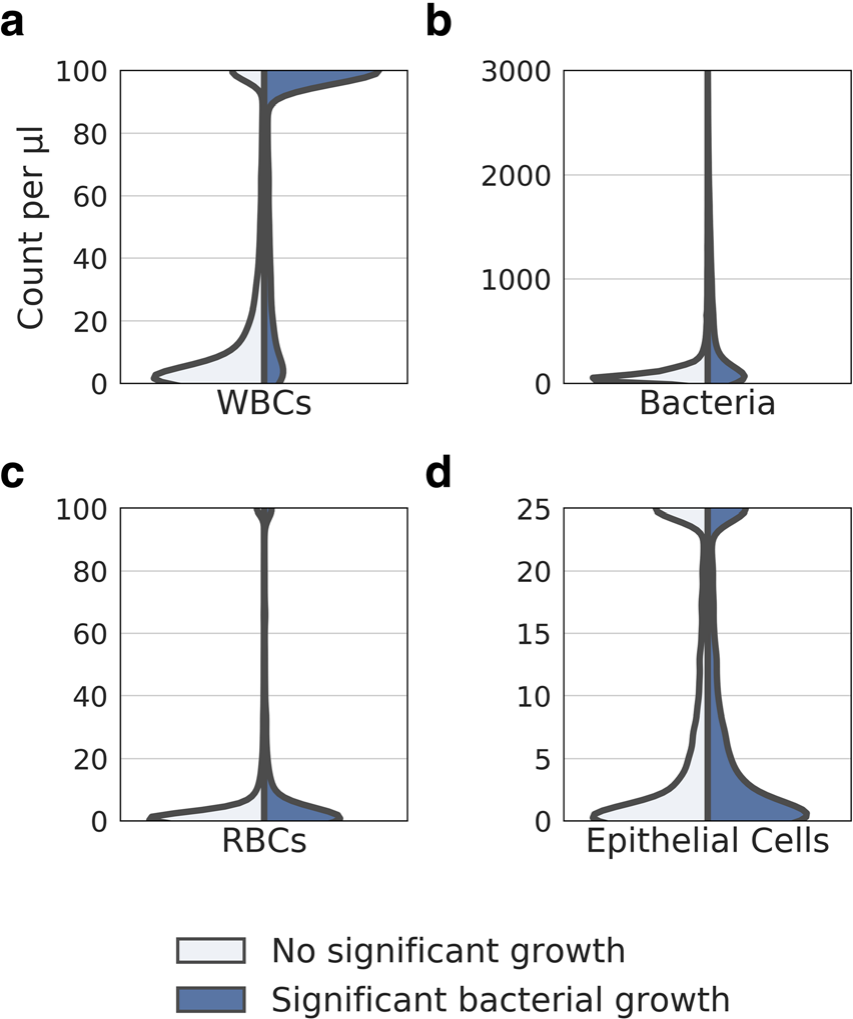
Distribution of microscopic cell count, for sample populations with and without significant bacterial growth on culture, for WBCs (**a**), bacterial cells (**b**), epithelial cells (**c**) and RBCs (**d**)

With regards to the distribution of automated microscopy cell counts, the patient population split into those with significant bacterial culture results and those without (Fig. 2). WBC counts demonstrated the greatest distinction between the population with significant culture results and the population without. Bacterial counts showed significant overlap between the two populations. Both were positively skewed, but to a greater extent for the population with significant culture results, which also displayed a lower kurtosis. A high WBC count was associated with an increase in significant bacterial growth, as were bacterial counts about 500 cells/μl. Low counts of WBC or bacteria were, however, not diagnostic of a negative culture result.

Patient groups were ranked and compared using the Chi-squared test for independence (implemented in Scikit-Learn feature selection module). Pyuria in the absence of RBCs, pregnancy, positive testing for nitrate, persistent/recurrent infection, and being an inpatient ranked the highest, showing they were the least likely to be independent of class, and therefore more valuable for classification. Additionally, gender, smell, and being pre-operative ranked higher than other categorical variables, such as whether the patient was immunocompromised (Additional file 1: Table S2). While these were the most highly ranked of the clinical indicators, they were not in themselves enough for classification of the bulk of patients due to the low numbers existing in the population. As an example, while being noted as being positive for nitrates was associated with a high probability of culturable bacteria (59·7%), this occurred in only 6·09% of the patients founds positive for bacterial culture. Hence, we examined the potential of heuristic and machine learning models that could include variables that were applicable to large numbers of patients.

### Performance of heuristic microscopy thresholds for predicting urine culture outcome

Given their strong association with positive bacterial culture, WBC counts and bacterial counts were chosen in combination to create a microscopy threshold for predicting culture outcome. Microscopy thresholds were compared using classification sensitivity, with 95% being chosen as the acceptable minimum. At the same time specificity, positive predictive value, negative predictive value, and the relative reduction in workload were calculated. By iterating over permutations from a range of WBC and bacterial counts, the effect of applied thresholds was simulated (Additional file 1: Tables S3 and S4).

Following simulation of microscopy thresholds, the optimum minimum thresholds for WBC and bacterial counts were found to be 30/μl and 100/μl, respectively. With these criteria it was simulated that there would be a 39·1% reduction in the number of samples needing culture and a classification sensitivity of 96·0 ± 0·1% (95% CI) for culture-positive urines (Table 3). Despite achieving the optimal sensitivity, the specificity of using a microscopy threshold was only 52·1 ± 0·4% (95% CI). The potential for an improved solution that reduced the number of false positive classifications resulted in exploration of supervised machine learning solutions incorporating additional variables.

### Integration of additional variables into machine learning algorithms

To measure the effectiveness of the machine learning algorithms, a Logistic Regression Classifier based on WBC and bacterial counts was used as a baseline. This algorithm exhibited similar performance to the use of microscopy threshold, as was to be expected as Logistic Regression classifiers are sensitive to non-linear relationships between independent and dependent variables; a condition suspected during exploratory data analysis.

The data exhibited a natural class imbalance in that only 27% of samples resulted in a positive culture outcome. Given that the purpose of this study was to create a screening method which would reduce the incidence of culture without compromising sensitivity, class weights were applied in such a way that false negative classifications were more heavily penalised than false positives. Initial class weights were chosen through grid search parameter optimisation and then adjusted manually to improve sensitivity. In the case of the neural network, resampling (without replacement) was used to eliminate class imbalance from the training data. Table 2 details the results of feature selection, performed using recursive feature elimination (RFE) to generate a list of optimal features; feature importance and AUC score in a Random Forest Classifier were used to eliminate features recursively. RFE suggested 16 optimal features (features with a ranking of 1).

**Table 2:** Feature selection by recursive feature elimination using a Random Forest Classifier. Feature importance is shown as well as the individual AUC score

	RFE Ranking	RF Feature Importance	Individual AUC^a^
WBC count	1	0·30	0·82
Bacterial count	1	0·30	0·71
Age	1	0·12	0·63
Epithelial cell count	1	0·07	0·49
RBC count	1	0·06	0·56
# of positive cultures to date	1	0·03	0·60
Pyuria, no RBCs	1	0·02	0·57
Pregnant	1	0·02	0·57
Inpatient	1	0·01	0·53
Gender	1	0·01	0·53
Persistent/recurrent infection	1	0·01	0·55
# of positive cultures month prior	1	0·009	0·53
Positive for nitrates	1	0·008	0·52
Renal inpatient/outpatient	1	0·005	0·50
Pre-operative patient	1	0·004	0·51
Acute kidney disease	1	0·003	0·50
Immunocompromised	2	0·002	0·50
# of positive cultures week prior	3	0·002	0·51
Multiple Sclerosis	4	0·001	0·50
Offensive smell	5	0·0007	0·50
Haematuria, no WBCs	6	0·0001	0·50

^a^Individual AUC score is calculated from a Logistic Regression classifier, where the feature in question is the sole independent variable

**Fig. 3:**
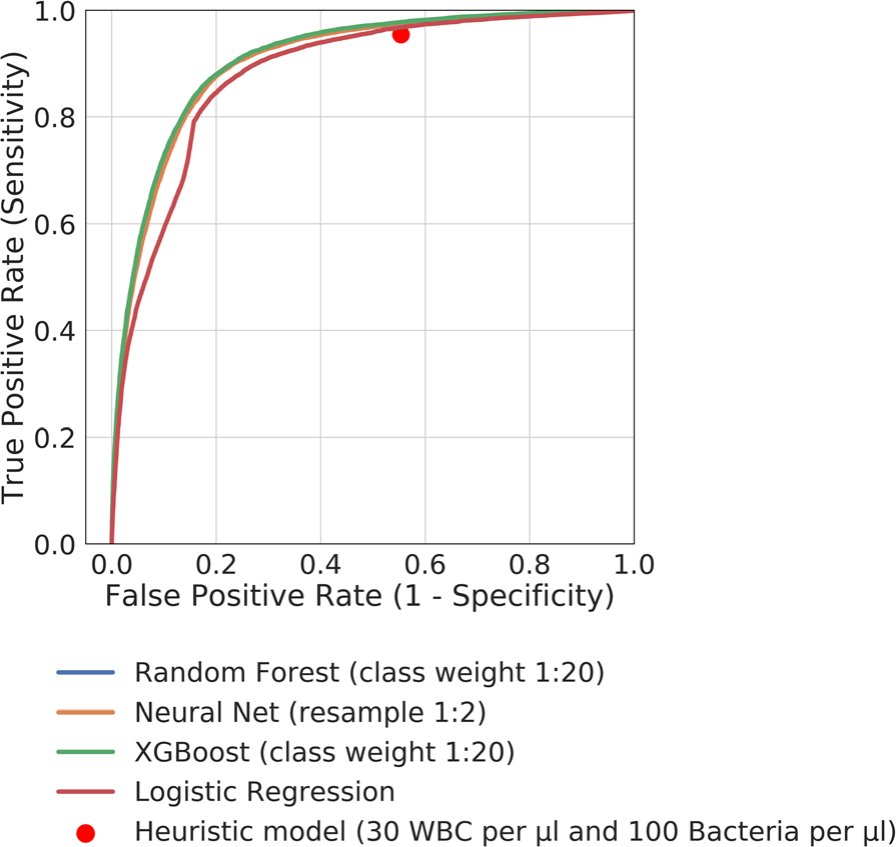
ROC curve for supervised machine learning models trained using the list of optimal features, in comparison to a Logistic Regression classifier trained solely using WBC count and bacterial count. Random Forest (class weight 1:20), AUC = 0·909; Neural Network (resample 1:2), AUC = 0·905; XGBoost (class weight 1:20), AUC = 0·910; Logistic Regression, AUC = 0·882. The red point indicates the performance of a heuristic model based on 30 WBC/μl and 100 bacteria/μl

The results of the supervised machine learning models when trained on the optimal features (those with an RFE ranking of 1) are shown in Table 3, with an accompanying ROC curve in Fig. 3. All machine learning algorithms outperformed the heuristic model (microscopy threshold of 30 WBC/μl and 100 bacteria/μl) in terms of accuracy. The Random Forest Classifier provided the best performance with a sensitivity of 95·95 ± 0·23% (95% CI) and a reduction in the number of necessary cultures by 47·58%. Cochran’s Q test found a statistically significant difference between models and post-hoc comparison to the heuristic model by McNemar’s test showed all models to be significantly different in terms of classification accuracy.

**Table 3:** Comparison of performance for heuristic and machine learning models tested on holdout data

Model Name	AUC Score	Accuracy (%)	*p*-value**	PPV	NPV	Sensitivity (%)			Specificity (%)	Relative Workload Reduction (%)
						All Patients	Pregnant	Children < 11 Yrs		
Heuristic model (30 WBC/μl or 100 bacteria/μl)		63·92	NA	42.73 [± 0.51]	97.01 [±0.28]	95·70 [± 0·15]	85·9 [± 0·72]	91·5 [± 0·92]	52·10 [± 0·36]	39·06 [± 0·38]
Random Forest (Class weight - 1:20)	0·908	71·96	< 0.001	40.47 [± 0.54]	97.67 [± 0.25]	95·95 [± 0·23]	70·5 [± 2·14]	89·8 [± 1·49]	63·40 [± 0·54]	47·58 [± 0·39]
Neural Network	0·906	85·00	< 0.001	71.70 [± 0.46]	90.18 [± 0.50]	74·03 [± 0·64]	27·6 [± 5·74]	69·3 [± 3·38]	89·09 [± 0·29]	71·98 [± 0·35]
Neural Network (with resampling*)	0·904	79·35	< 0.001	57.66 [± 0.74]	95.54 [± 0.19]	90·60 [± 0·35]	56·6 [± 3·43]	84·8 [± 2·04]	75·16 [± 0·44]	57·33 [± 0·38]
XGBoost (Class weight - 1:20)	0·910	65·68	< 0.001	44.05 [± 0.74]	97.77 [± 0.13]	96·70 [± 0·18]	77·1 [± 1·65]	93·1 [± 1·13]	54·14 [± 0·61]	40·36 [± 0·38]

[95% Confidence Interval]

*Resampling (without replacement) at a ratio of 2:1 for positive samples to offset class imbalance

** *p*-values obtained by comparison to heuristic model by McNemar test

### Classification of pregnant patients

**Fig. 4:**
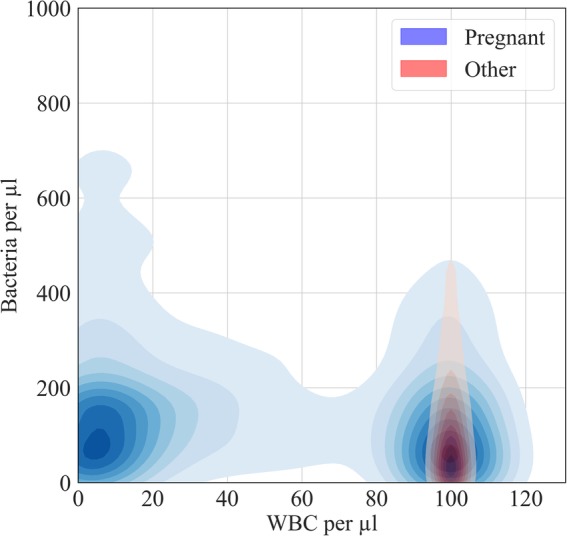
Bivariate kernel density estimates for samples with significant bacterial growth on culture. Pregnant patients exhibit a greater proportion of culture positive samples with a reduced white cell count despite an increased bacterial count. It should be noted that the lowest contour is not shown for visual clarity

When observing the classification sensitivity for different patient demographics, it was noted that the sensitivity for pregnant patients was in the range of 56–86% across all models, below the sensitivity for the general population. Asymptomatic bacteriuria is a condition known to occur in 2–10% of pregnancies and is associated with adverse outcomes such as increased risk of preterm birth, low birth weight, and perinatal mortality [26]. Figure 4 compares the kernel density estimate for WBC and bacterial counts, where there was significant bacterial growth on culture, for pregnant patients and all other patients. For pregnant patients there was a greater prevalence of samples with increased bacterial count in the absence of WBCs, which may explain the poor classification sensitivity in comparison to other patient groups.

**Fig. 5:**
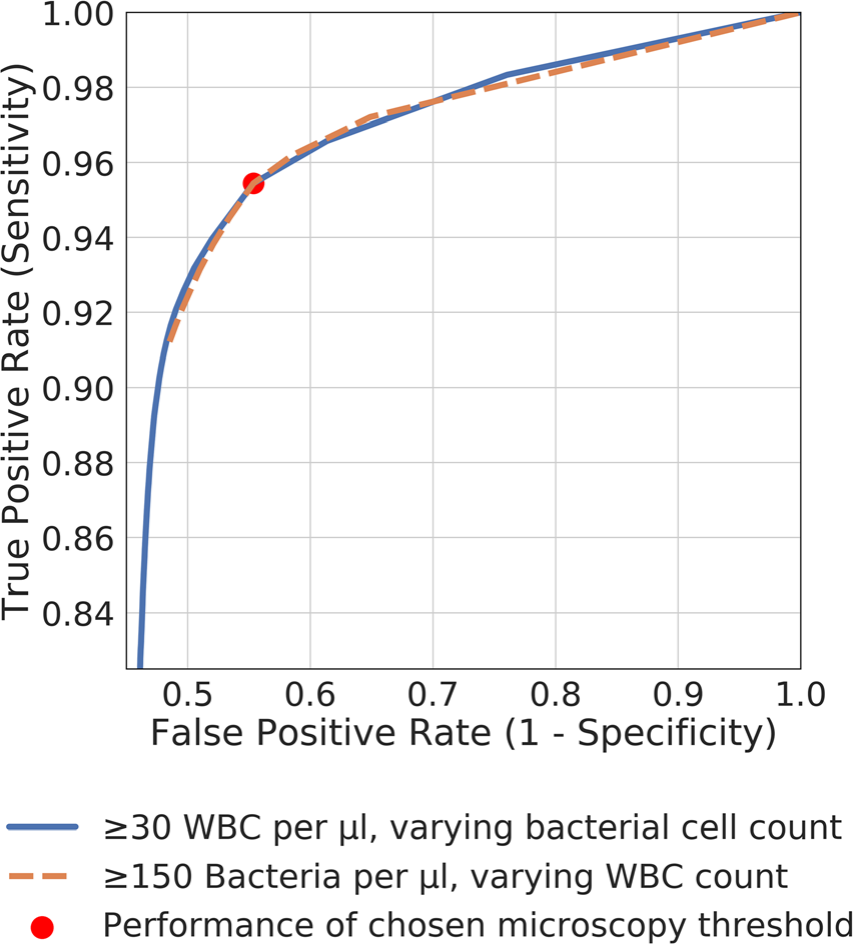
ROC curve for varying WBC count and varying bacterial count, calculated after the removal of pregnant patients and children less than 11 years old. The red point indicates the combined threshold chosen for optimal performance

Considering that all samples from pregnant patients and children under 11 years of age should be cultured routinely according to the recommendations by the UK Standards for Microbiology Investigations [2], the heuristic model was re-examined and microscopy thresholds analysed with those patients removed (Table 4). The new optimal microscopy threshold was found to be 30 WBC/μl and 150 bacteria/μl. This threshold performed with a sensitivity of 95·0 ± 0·1% (95% CI) and a relative workload reduction of 33·7% (Table 4, Fig. 5). Due to the considerable cost savings without compromising diagnostic performance, this model went on to be implemented into clinical practice at the Severn Pathology service in Bristol, UK.

**Table 4:** Comparison of performance for heuristic and machine learning models with additional consideration for pregnant patients and children less than 11 years old

Model Name	AUC Score	Accuracy (%)	*p*-value***	PPV	NPV	Sensitivity (%)	Specificity (%)	Relative Workload Reduction (%)
Removal of pregnant patients and children (< 11 yrs)*								
Heuristic mode (30 WBC/μl or 150 bacteria/μl)		58·40	NA	39.14 [± 0.73]	96.29 [± 0.17]	95·4 [± 0·14]	44·60 [± 0·34]	33·74 [± 0·39]
Random Forest (Class weight - 1:8)	0·920	77·09	< 0.001	53.25 [± 0.50]	97.46 [± 0.26]	95·2 [± 0·26]	68·79 [± 0·58]	38·92 [± 0·42]
Combined XGBoost**								
Pregnant patients	0·828	26·94				94·6 [± 0·56]	26·84 [± 1·88]	25·29 [± 0·92]
Children (< 11 yrs)	0·913	62·00				94·8 [± 0·88]	55·00 [± 2·12]	46·24 [± 1·48]
Pregnant patients	0·894	71·65				95·3 [± 0·24]	60·93 [± 0·65]	43·38 [± 0·41]
Combined performance	0.749	65·65	< 0.001	47.64 [± 0.51]	97.14 [± 0.28]	95·2 [± 0·22]	60·93 [± 0·60]	41·18 [± 0·39]

[95% Confidence Interval]

*Pregnant patients and children (< 11 yrs) are not included in the classification process. It is assumed that all patients in these populations will receive culture and this is reflected in the reported relative workload reduction

** Independent classification algorithms trained and tested on stratified patient populations

*** *p*-values obtained by comparison to heuristic model by McNemar test

In response to this finding, machine learning algorithms were revisited with the removal of pregnant patients and children less than 11 years old from the classification process. Since the Random Forest classifier provided the best performance previously, a new implementation of this algorithm was trained on a randomly selected cohort of 70% of the remaining data; 30% was kept as holdout for evaluation of model performance. Parameter optimisation was performed using grid search with a reduced class weight of 1:8 for positive culture when considering samples other than pregnant patients. As shown in Table 4, a Random Forest Classifier that considers additional variables could achieve a specificity of 68·8% compared with the specificity of the heuristic model of 44·6%. However, given that samples from pregnant women and children under 11 together comprise 29.2% of samples entering the pipeline, the overall, workload reduction only improved by around 4%.

**Fig. 6:**
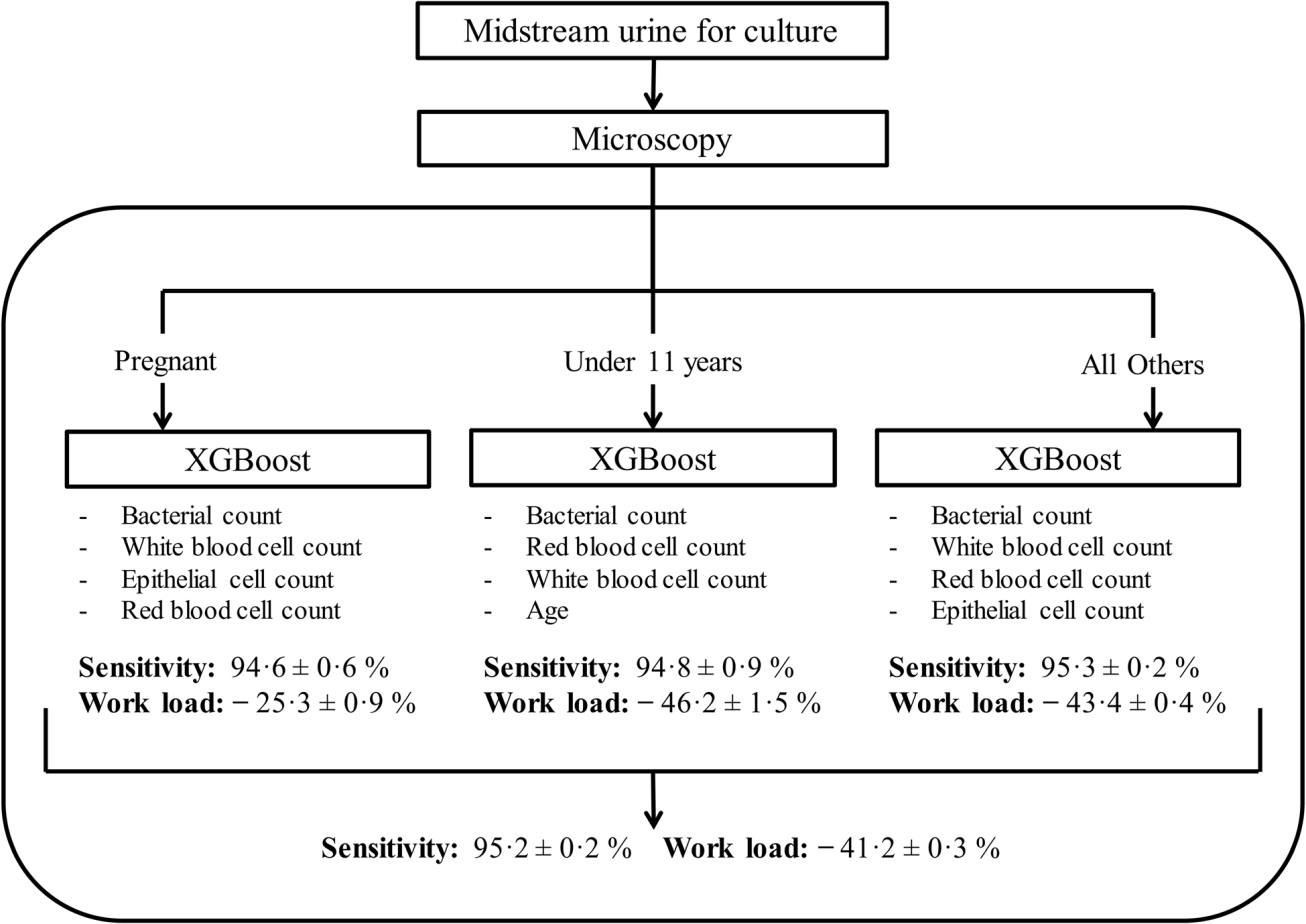
Performance of the optimal model, with independent classification algorithms for stratified patient groups, as predicted from validation data. The top four features are ranked by average feature importance for all decision trees in the model. Performance is shown as sensitivity ±95% confidence interval

The alternative approach was to separate pregnant patients and children from all other samples, creating three separate datasets. Training and validation data was generated for each dataset following the same methodology as previously described. Three independent XGBoost models were trained, one for each dataset. XGBoost is a resource efficient algorithm that exhibits greater computational performance [15]. For this reason, combined with good classification performance in prior experiments, it was chosen over all other machine learning models going forward. The algorithms were trained independently of one another and evaluated on holdout data from their separate populations (pregnant, children, and everyone else). Classification sensitivity for pregnant patients, children, and samples from all other patients was 95·4%, 94·9% and 95·3% respectively. When tested on the validation data, the combined workload reduction from the three independent models was 41.2%, a significant improvement over the performance of the heuristic model. This combination of XGBoost models gives optimal performance in terms of classification sensitivity and relative workload reduction and is summarised in Fig. 6.

## Discussion

To our knowledge, there are no other observational studies of this magnitude for the study of urine analysis for the diagnosis of UTIs. Most previous studies with the objective of predicting urine culture based on variables generated from sediment analysis, flow cytometry, and/or dip-stick testing have been controlled studies of a few hundred patients, with little consistency in the inclusion criteria [3, 4, 6, 13, 27–29]. Prior efforts to establish a heuristic model based on microscopy thresholds generated conflicting results. Falbo et al. [4] and Inigo et al. [3] reported a sensitivity and specificity in the range of 96–98% and 59–63% respectively, with microscopy thresholds on sample populations of less than 1000. Both studies reported an optimum WBC count (cells/μl) of 18 but differing bacterial counts (44/μl and 97/μl respectively). Variation in results between the two studies is likely to be due to small sample size. It should also be noted that neither study adjusted for pregnant patients or children under the age of 11, and the sensitivity of classification for vulnerable demographics was not shared. Additionally, greater than 50% of samples in the study by Inigo et al. originated from inpatients and both studies included specimens from catheterised patients [3, 4]. In contrast to those findings, Sterry-Blunt et al. [6] reported from a study of 1411 samples that the highest achievable negative predictive value when using white blood cell and bacterial count thresholds was 89·1% and concluded that the SediMAX should not be used as a screening method prior to culture.

The use of flow cytometry for urine analysis prior to culture has been gaining popularity as a replacement to automated urine microscopy and shows good performance in the literature. Multiple studies have now shown that the use of flow cytometry with optimised cell count thresholds provides greater specificity without compromising sensitivity when classifying urine samples [3, 27, 30–32]. Future work should investigate the benefit of using machine learning algorithms that include cellular counts generated using flow cytometry methods as opposed to automated microscopy.

Taking advantage of recent developments in ‘big data’ technologies, our observational study analysed data representing an entire year of urine analysis at a large pathology service that covers sample processing for multiple hospitals as well as the community in the Bristol/Bath region in the Southwest of the UK. To our knowledge there have been no attempts to apply machine learning techniques for the purpose of predicting urine culture outcome in a laboratory setting. Taylor et al. [5] applied supervised machine learning to predict UTIs in symptomatic emergency department patients. An observational study of 80,387 adult patients, using 211 variables of both clinical and laboratory data, was used to develop 6 machine learning algorithms that were then compared to documentation of UTI diagnosis and antibiotic administration. The study concluded that the XGBoost algorithm outperformed all other classifiers and when compared to the documented diagnosis, application of the algorithm would approximate to 1 in 4 patients being re-categorised from false positive to true negative, and 1 in 11 patients being re-categorised from false negative to true positive. The XGBoost algorithm presented has similar performance to the one trained on our dataset, with an AUC score of 0·904. The sensitivity was poor however, at 61·7%, and a corresponding specificity of 94·9%. It is suspected that the difference in sensitivity between our models is the result of the application of class weights. Taylor et al. [5] did not disclose any parameter tuning of this sort and the sensitivity reported was likely a result of class imbalance (only 23% of their training consists of positive samples). Here, we applied class weights to direct a classification algorithm that favored a high sensitivity and met the criteria expected of a screening test.

Our study made considerations for the high risk groups of pregnant patients and children under the age of 11, with the objective to generate a predictive algorithm that would conform to the UK standards of microbiological investigations. We also classified patients into groups based on identification of key words in clinical details provided by the requesting clinician. Although methods were put into place to increase the accuracy of these classifications (employment of a Levenshtein distance algorithm and consolidation of clinical details from patients with multiple samples) the free-form nature of the notes means that key words would not always be included even when applicable. This has likely led to an underestimation of some groups, but it is possible that this may be addressed in future by more advanced text mining of clinical notes, such as the use of deep learning techniques that can classify patients into medical subdomains, as shown successfully by Weng et al. [33].

In our dataset, when observing samples that have generated a positive bacterial culture, there is a clear difference in the distribution of white cell counts in pregnant patients compared to all other patients. The changes in the immune response during pregnancy are not fully understood but it is agreed that modulation of the immune system is significantly changed [26]. This could explain the differences observed in our dataset, but we must also consider the contribution from the screening for asymptomatic bacteriuria in pregnant patients during the middle trimester. Although asymptomatic bacteriuria is cited as an associated with adverse outcomes [26, 27], a randomised control study of 5132 pregnant patients in the Netherlands reported a low risk of pyelonephritis in untreated asymptomatic bacteriuria, question the use of such screening [7].

Our study demonstrates the power of machine learning algorithms in defining critical variables for clinical diagnosis of suspected UTIs. Given increasing demand due to ageing populations in most developed and developing countries, radical change is needed to improve cost efficiency and optimise capacity in diagnostic laboratories. At a time when antimicrobial resistance is dramatically on the rise amongst Gram-negative bacteria, including the two most common urinary pathogens, *E. coli* and *Klebsiella pneumoniae*, any significant reduction in inappropriate sample processing will have a positive impact on the turn-around time for clinically relevant infection and improve time to appropriate therapy and antimicrobial stewardship.

Extrapolating our estimated workload reduction on a national scale, the savings made in reduction of purchases of culture agar alone (without considering the time cost and additional expenses involved in performing bacterial culture), the implementation of the three XGBoost algorithms as described in Fig. 6 would result in savings of £800,000–5 million per year across the UK (estimates are based on local purchasing data and online sources [34]).

There are several limitations of this study. Firstly, the retrospective nature of the study makes it difficult to clarify some of the details such as potential mis-labelling of samples. However, the use of over 200,000 samples archived with a state-of-the-art LIMS system should ensure the data are relatively robust to random individual errors in labelling. Secondly, the clinical details provided by the requesting clinicians were relatively sparse. This is true for most diagnostic requests in a busy and publicly-funded hospital, where doctors must prioritise their limited time. Hence, the dataset represents the “real life” scenario. Thirdly, it should be remembered that the outcome we have studied is a culture predictability rather than clinical/therapeutic outcome.

## Conclusion

The work presented here shows that supervised machine learning models can be of significant utility in predicting whether urine samples are likely to require bacterial culture. We also highlight the importance of identifying vulnerable patient groups and propose a combination of independent algorithms targeted at each group separately. When using a methodology such as this, we demonstrate a potential reduction in culture workload of around 41% while detecting 95·2 ± 0·22% of culture positive samples successfully. This could potentially improve service efficiency at a time when demand is surpassing the resources of public healthcare providers.

## Additional files

Additional file 1: **Table S1.** Patient groups of significant clinical interest when investigating the presence of UTI, along with corresponding keywords included in the Levenshtein distance algorithm used to classify samples. **Table S2.** Comparison of categorical variables using Chi-squared statistic (all *p*-values < 0·0001). **Table S3a.** Classification sensitivity (%) for simulation of microscopy thresholds on retrospective data (including pregnant patients and children < 11 years in classification). **Table S3b.** Relative workload reduction (%) for simulation of microscopy thresholds on retrospective data (including pregnant patients and children < 11 years in classification). **Table S4a.** Classification sensitivity (%) for simulation of microscopy thresholds on retrospective data after removal of pregnant patients and children < 11 yrs. who will receive culture regardless of microscopy cell count. **Table S4b.** Relative workload reduction (%) for simulation of microscopy thresholds on retrospective data after removal of pregnant patients and children < 11 years who will receive culture regardless of microscopy cell count. (DOCX 31 kb)

Additional file 2: **Figure S1.** Pre-processing steps prior to study of microscopy thresholds and machine learning models. (PNG 57 kb)

Additional file 3: **Figure S2.** Formula for calculation of sensitivity, specificity, and accompanying confidence intervals. 1,96 is the probit for a target error rate of 0.05. (PNG 975 kb)

Additional file 4: **Figure S3.** Age distribution for samples received from male (a) and female (b) patients. *, 51% of patients between the age of 20 and 40 were pregnant, compared to 1·8% of patients outside this age range. (TIF 33750 kb)

## Data Availability

The datasets used and/or analysed during the current study are available from the corresponding author on reasonable request.

## Abbreviations

AUC: Area Under Curve
LIMS: Laboratory Information Management System
NHS: National Health Service
NLTK: Natural Language Toolkit
RBC: Red Blood Cell
RFE: Recurrent Feature Elimination
ROC: Receiver Operating Characteristic
UTI: Urinary Tract Infection
WBC: White Blood Cell
XGBoost: Extreme Gradient Boosting
