# Supplementary material for: Using artificial intelligence to reduce diagnostic workload without compromising detection of urinary tract infections
Source: BMC Med Inform Decis Mak. 2019 Aug 23;19:171. doi: 10.1186/s12911-019-0878-9 (PMC6708133; doi:10.1186/s12911-019-0878-9)
Supplement: Supplementary file 1 — Table S1. Patient groups of significant clinical interest when investigating the presence of UTI, along with corresponding keywords included in the Levenshtein distance algorithm used to classify samples. Table S2. Comparison of categorical variables using Chi-squared statistic (all p-values < 0·0001). Table S3a. Classification sensitivity (%) for simulation of microscopy thresholds on retrospective data (including pregnant patients and children < 11 years in classification). Table S3b. Relative workload reduction (%) for simulation of microscopy thresholds on retrospective data (including pregnant patients and children < 11 years in classification). Table S4a. Classification sensitivity (%) for simulation of microscopy thresholds on retrospective data after removal of pregnant patients and children < 11 yrs. who will receive culture regardless of microscopy cell count. Table S4b. Relative workload reduction (%) for simulation of microscopy thresholds on retrospective data after removal of pregnant patients and children < 11 years who will receive culture regardless of microscopy cell count. (DOCX 31 kb) [file 12911_2019_878_MOESM1_ESM.docx]

| **Patient group** | **Keywords for search algorithm** |
| --- | --- |
| Pregnant | 'pregnant','pregnancy','/40','pregn','cyesis','booking', 'antenatal' |
| Acute kidney infection | 'pyelonephritis','kidney infection', ‘acute kidney’ |
| Renal inpatient/outpatient | 'renal', 'urology', ‘neurogenic’, ‘ckd’, ‘chronic kidney’ |
| Persistent/recurrent infection | 'persistent','recurrent' |
| Multiple Sclerosis | 'ms','Multiple sclerosis', 'sclerosis' |
| Pre-operative patient | 'pre-op','preop', 'pre-operative', 'preoperative' |
| Immunocompromised/Oncology | 'immunocompromised','chemotherapy','cancer','carcinoma','chemo','HIV','AIDS', 'neutropenic' |

**Additional file 1: Table S1.** Patient groups of significant clinical interest when investigating the presence of UTI, along with corresponding keywords included in the Levenshtein distance algorithm used to classify samples.

|  | Chi-Squared Statistic |
| --- | --- |
| Multiple Sclerosis | 4·18 |
| Chronic urological disease | 5·82 |
| Immunocompromised | 17·20 |
| Acute kidney disease | 31·98 |
| Haematuria, no WBCs | 81·52 |
| Smell | 105·87 |
| Pre-operative | 136·76 |
| Gender | 293·06 |
| Inpatient | 893·07 |
| Persistent/recurrent infection | 2,633·04 |
| Positive for nitrates | 3,152·13 |
| Pregnant | 5,719·68 |
| Pyuria, no RBCs | 7,783·40 |

**Additional file 1: Table S2.** Comparison of categorical variables using Chi-squared statistic (all p-values < 0·0001)

|  | Bacterial count (per μl) | | | | | | | | | | | | | | |
| --- | --- | --- | --- | --- | --- | --- | --- | --- | --- | --- | --- | --- | --- | --- | --- |
| WBC count (per μl) | 10 | 20 | 30 | 40 | 50 | 60 | 70 | 80 | 90 | 100 | 110 | 120 | 130 | 140 | 150 |
| 10 | 99·59 | 99·35 | 99·02 | 98·71 | 98·44 | 98·14 | 97·85 | 97·57 | 97·35 | 97·15 | 96·98 | 96·81 | 96·65 | 96·51 | 96·36 |
| 20 | 99·48 | 99·19 | 98·81 | 98·43 | 98·08 | 97·68 | 97·30 | 96·90 | 96·58 | 96·30 | 96·04 | 95·78 | 95·57 | 95·38 | 95·18 |
| 30 | 99·43 | 99·12 | 98·70 | 98·30 | 97·88 | 97·41 | 96·96 | 96·44 | 96·05 | 95·71 | 95·38 | 95·07 | 94·82 | 94·56 | 94·33 |
| 40 | 99·36 | 99·02 | 98·55 | 98·07 | 97·55 | 96·99 | 96·46 | 95·84 | 95·37 | 94·97 | 94·57 | 94·20 | 93·89 | 93·60 | 93·32 |
| 50 | 99·30 | 98·95 | 98·44 | 97·92 | 97·33 | 96·71 | 96·09 | 95·39 | 94·87 | 94·40 | 93·96 | 93·53 | 93·18 | 92·85 | 92·52 |
| 60 | 99·25 | 98·89 | 98·34 | 97·77 | 97·13 | 96·45 | 95·77 | 95·00 | 94·40 | 93·89 | 93·39 | 92·92 | 92·53 | 92·16 | 91·80 |
| 70 | 99·19 | 98·80 | 98·21 | 97·61 | 96·90 | 96·17 | 95·44 | 94·61 | 93·95 | 93·38 | 92·83 | 92·32 | 91·91 | 91·51 | 91·11 |
| 80 | 99·18 | 98·78 | 98·17 | 97·55 | 96·80 | 96·02 | 95·24 | 94·35 | 93·65 | 93·03 | 92·45 | 91·89 | 91·46 | 91·02 | 90·60 |
| 90 | 99·16 | 98·75 | 98·12 | 97·47 | 96·67 | 95·86 | 95·02 | 94·09 | 93·34 | 92·68 | 92·06 | 91·48 | 91·01 | 90·56 | 90·11 |
| 100 | 99·13 | 98·71 | 98·07 | 97·40 | 96·56 | 95·70 | 94·82 | 93·85 | 93·06 | 92·35 | 91·72 | 91·11 | 90·61 | 90·14 | 89·67 |

**Additional file 1: Table S3a.** Classification sensitivity (%) for simulation of microscopy thresholds on retrospective data (including pregnant patients and children <11 years in classification).

|  | Bacterial count (per μl) | | | | | | | | | | | | | | | |
| --- | --- | --- | --- | --- | --- | --- | --- | --- | --- | --- | --- | --- | --- | --- | --- | --- |
| WBC count (per μl) | 10 | 20 | 30 | 40 | 50 | 60 | 70 | 80 | 90 | 100 | 110 | 120 | 130 | 140 | 150 |  |
| 10 | 2·13 | 6·57 | 11·87 | 16·61 | 20·54 | 24·00 | 26·62 | 28·82 | 30·57 | 31·98 | 33·19 | 34·30 | 35·17 | 35·88 | 36·51 |  |
| 20 | 2·34 | 6·98 | 12·76 | 18·11 | 22·67 | 26·76 | 29·91 | 32·67 | 34·90 | 36·73 | 38·30 | 39·77 | 40·92 | 41·90 | 42·75 |  |
| 30 | 2·45 | 7·19 | 13·17 | 18·79 | 23·67 | 28·06 | 31·48 | 34·53 | 37·00 | 39·06 | 40·84 | 42·51 | 43·83 | 44·96 | 45·93 |  |
| 40 | 2·53 | 7·32 | 13·41 | 19·21 | 24·31 | 28·89 | 32·51 | 35·77 | 38·40 | 40·61 | 42·52 | 44·33 | 45·74 | 46·97 | 48·02 |  |
| 50 | 2·58 | 7·40 | 13·55 | 19·46 | 24·69 | 29·41 | 33·17 | 36·56 | 39·31 | 41·63 | 43·63 | 45·53 | 47·02 | 48·32 | 49·44 |  |
| 60 | 2·62 | 7·45 | 13·66 | 19·66 | 24·99 | 29·83 | 33·71 | 37·20 | 40·05 | 42·45 | 44·53 | 46·51 | 48·06 | 49·43 | 50·60 |  |
| 70 | 2·68 | 7·54 | 13·81 | 19·87 | 25·28 | 30·21 | 34·16 | 37·74 | 40·68 | 43·15 | 45·29 | 47·33 | 48·92 | 50·34 | 51·55 |  |
| 80 | 2·70 | 7·58 | 13·87 | 19·98 | 25·46 | 30·46 | 34·49 | 38·13 | 41·12 | 43·65 | 45·85 | 47·93 | 49·56 | 51·02 | 52·26 |  |
| 90 | 2·71 | 7·60 | 13·92 | 20·06 | 25·60 | 30·67 | 34·75 | 38·45 | 41·49 | 44·07 | 46·31 | 48·43 | 50·09 | 51·58 | 52·85 |  |
| 100 | 2·73 | 7·63 | 13·97 | 20·15 | 25·74 | 30·86 | 35·00 | 38·74 | 41·83 | 44·44 | 46·72 | 48·87 | 50·57 | 52·08 | 53·37 |  |

**Additional file 1: Table S3b.** Relative workload reduction (%) for simulation of microscopy thresholds on retrospective data (including pregnant patients and children <11 years in classification).

|  | Bacterial count (per μl) | | | | | | | |
| --- | --- | --- | --- | --- | --- | --- | --- | --- |
| WBC count (per μl) | 100 | 110 | 120 | 130 | 140 | 150 | 160 | 170 |
| 30 | 96·58 | 96·31 | 96·05 | 95·84 | 95·64 | 95·45 | 95·20 | 94·57 |
| 40 | 95·89 | 95·57 | 95·26 | 95·00 | 94·76 | 94·53 | 94·25 | 93·54 |
| 50 | 95·38 | 95·02 | 94·66 | 94·36 | 94·08 | 93·81 | 93·51 | 92·71 |
| 60 | 94·91 | 94·49 | 94·10 | 93·76 | 93·44 | 93·15 | 92·81 | 91·97 |
| 70 | 94·45 | 93·99 | 93·56 | 93·21 | 92·86 | 92·53 | 92·17 | 91·20 |
| 80 | 94·13 | 93·64 | 93·17 | 92·80 | 92·42 | 92·06 | 91·68 | 90·66 |

**Additional file 1: Table S4a.** Classification sensitivity (%) for simulation of microscopy thresholds on retrospective data after removal of pregnant patients and children <11yrs who will receive culture regardless of microscopy cell count.

|  | Bacterial count (per μl) | | | | | | | |
| --- | --- | --- | --- | --- | --- | --- | --- | --- |
| WBC count (per μl) | 100 | 110 | 120 | 130 | 140 | 150 | 160 | 170 |
| 30 | 29·07 | 30·30 | 31·44 | 32·32 | 33·08 | 33·74 | 34·33 | 35·39 |
| 40 | 30·33 | 31·66 | 32·90 | 33·87 | 34·70 | 35·41 | 36·06 | 37·20 |
| 50 | 31·18 | 32·59 | 33·91 | 34·93 | 35·82 | 36·58 | 37·27 | 38·46 |
| 60 | 31·88 | 33·35 | 34·72 | 35·80 | 36·74 | 37·55 | 38·27 | 39·51 |
| 70 | 32·47 | 33·99 | 35·41 | 36·52 | 37·50 | 38·34 | 39·09 | 40·41 |
| 80 | 32·89 | 34·46 | 35·92 | 37·06 | 38·06 | 38·93 | 39·70 | 41·05 |

**Additional file 1: Table S4b.** Relative workload reduction (%) for simulation of microscopy thresholds on retrospective data after removal of pregnant patients and children <11 years who will receive culture regardless of microscopy cell count.
