# Supplementary figures and images for: Using artificial intelligence to reduce diagnostic workload without compromising detection of urinary tract infections
Source: BMC Med Inform Decis Mak. 2019 Aug 23;19:171. doi: 10.1186/s12911-019-0878-9 (PMC6708133; doi:10.1186/s12911-019-0878-9)

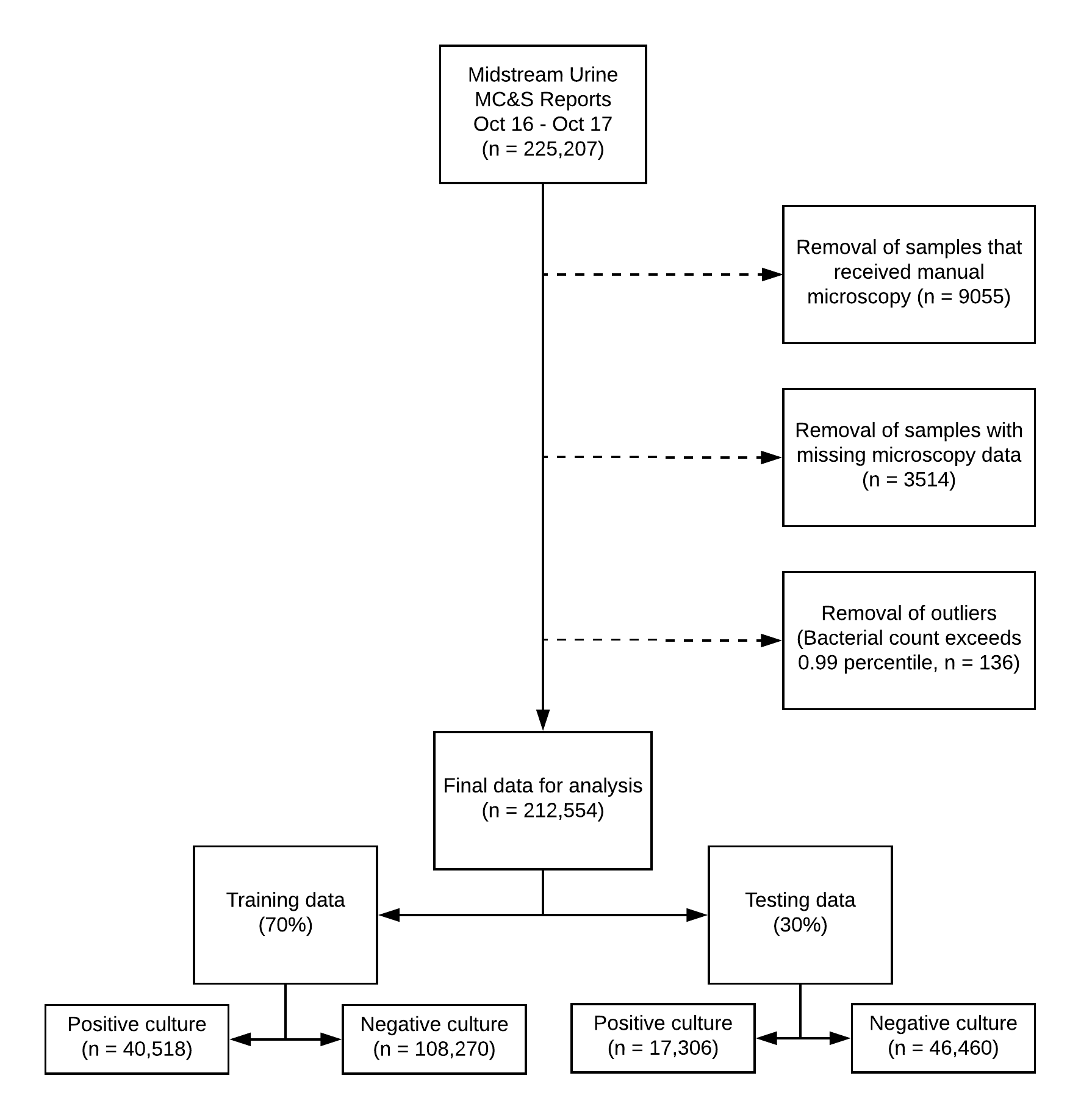

Supplement: Supplementary file 2 — Figure S1. Pre-processing steps prior to study of microscopy thresholds and machine learning models. (PNG 57 kb) [file 12911_2019_878_MOESM2_ESM.png]

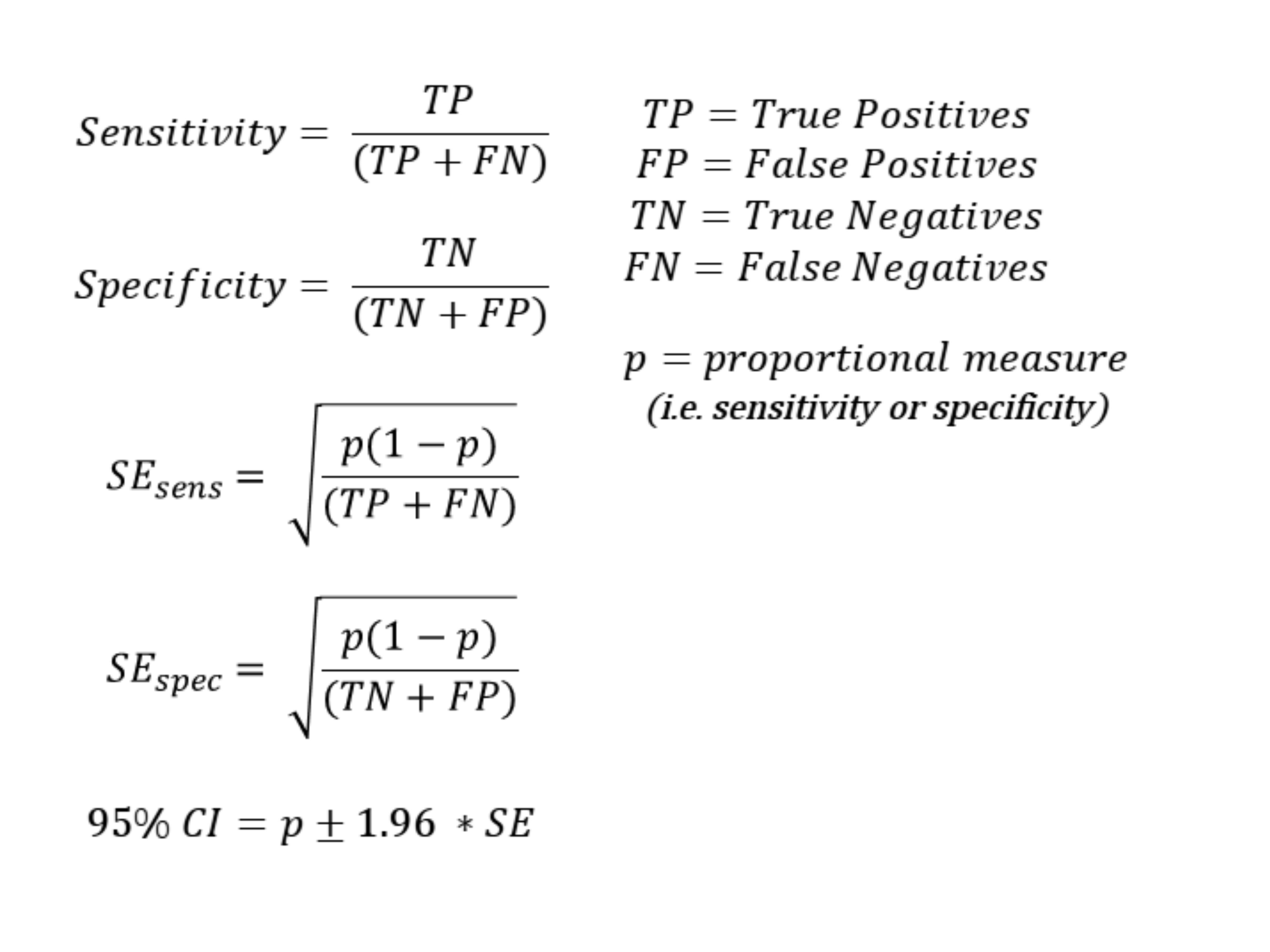

Supplement: Supplementary file 3 — Figure S2. Formula for calculation of sensitivity, specificity, and accompanying confidence intervals. 1,96 is the probit for a target error rate of 0.05. (PNG 975 kb) [file 12911_2019_878_MOESM3_ESM.png]

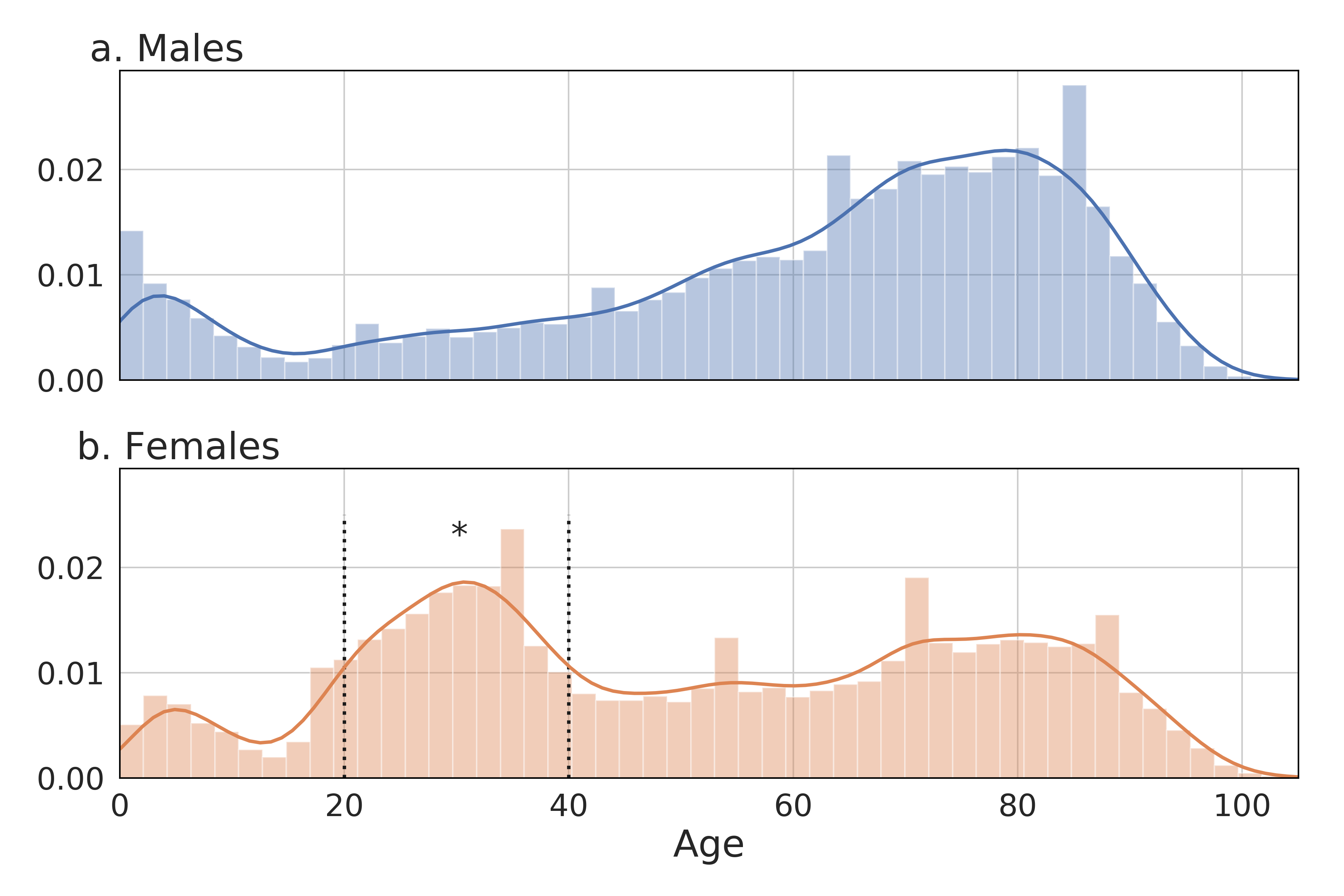

Supplement: Supplementary file 4 — Figure S3. Age distribution for samples received from male (a) and female (b) patients. *, 51% of patients between the age of 20 and 40 were pregnant, compared to 1·8% of patients outside this age range. (TIF 33750 kb) [file 12911_2019_878_MOESM4_ESM.tif]
